# Understanding and restoring dopaminergic function in fibromyalgia patients using a mindfulness-based psychological intervention: a [18F]-DOPA PET study. Study protocol for the FIBRODOPA study—a randomized controlled trial

**DOI:** 10.1186/s13063-021-05798-1

**Published:** 2021-12-01

**Authors:** K. Ledermann, R. von Känel, C. Berna, H. Sprott, M. Burckhardt, J. Jenewein, E. L. Garland, C. Martin-Sölch

**Affiliations:** 1 Department for Consultation-Liaison Psychiatry and Psychosomatic Medicine, University of Zurich, Zurich, Switzerland; 2 Department of Psychology, Chair of Clinical and Health Psychology, I-Reach Lab, University of Fribourg, Rue de Faucigny 2, 1700 Fribourg, Switzerland; 3 Center for Integrative and Complementary Medicine, Division of Anesthesiology, Lausanne University Hospital, University of Lausanne, Lausanne, Switzerland; 4 University of Zurich, Arztpraxis Hottingen Zurich, Zürich, Switzerland; 5 Medizinische Universität Graz, Universitätsklinik für medizinische Psychologie und Psychotherapie, Graz, Austria; 6 College of Social Work, Center on Mindfulness and Integrative Health Intervention Development, University of Utah, Salt Lake City, USA

**Keywords:** Fibromyalgia, Mindfulness-Oriented Recovery Enhancement, Dopamine, Reward, F-18 DOPA PET, fMRI

## Abstract

**Background:**

Fibromyalgia (FM) is a very prevalent and debilitating chronic pain disorder that is difficult to treat. Mindfulness-based techniques are regarded as a very promising approach for the treatment of chronic pain and in particular FM. The Mindfulness-Oriented Recovery Enhancement (MORE) intervention, a mindfulness-based group intervention, has shown beneficial effects in opioid-treated chronic pain patients, including reduced pain severity, functional interference, and opioid dosing, by restoring neurophysiological and behavioral responses to reward. The first evidence for a hypodopaminergic state and impaired reward processing in FM has been reported. However, little is known about its impact on dopamine (DA) function and in particular with regard to DA responses to monetary reward in FM. The aim of the present study protocol is to evaluate if MORE is able to restore the DA function in FM patients, in particular with regard to the DA responses to reward, and to reduce pain and mood complaints in FM.

**Methods:**

The present study is a multi-center interventional RCT with 3 time points: before the intervention, after completion of the intervention, and 3 months after completion of the intervention. Sixty-four FM patients will be randomly assigned to either the MORE intervention (*N* = 32) or a non-intervention control group (*N* = 32). Additionally, a comparison group of healthy women (*N* = 20) for PET measures will be enrolled and another group of healthy women (*N* = 15) will do the ambulatory assessments only. The MORE intervention consists of eight 2-h-long group sessions administered weekly over a period of 8 weeks. Before and after the intervention, FM participants will undergo [18F] DOPA positron emission tomography (PET) and functional MR imaging while performing a reward task. The primary outcome will be endogeneous DA changes measured with [18F] DOPA PET at baseline, after the intervention (after 8 weeks for the non-intervention control group), and at 3 months’ follow-up. Secondary outcomes will be (1) clinical pain measures and FM symptoms using standardized clinical scales; (2) functional brain changes; (3) measures of negative and positive affect, stress, and reward experience in daily life using the ambulatory assessment method (AA); and (4) biological measures of stress including cortisol and alpha-amylase.

**Discussion:**

If the findings of this study confirm the effectiveness of MORE in restoring DA function, reducing pain, and improving mood symptoms, MORE can be judged to be a promising means to improve the quality of life in FM patients. The findings of this trial may inform health care providers about the potential use of the MORE intervention as a possible non-pharmacological intervention for FM.

**Trial registration:**

ClinicalTrials.govNCT 04451564. Registered on 3 July 2020. The trial was prospectively registered.

## Administrative information

Note: the numbers in curly brackets in this protocol refer to SPIRIT checklist item numbers. The order of the items has been modified to group similar items (see).

**Table:** 

Title {1}	Understanding and restoring dopaminergic function in Fibromyalgia patients using a mindfulness-based psychological intervention: A [18F]-DOPA PET study Study protocol for the FIBRODOPA study- a randomized controlled trial
Trial registration {2a and 2b}.	The protocol was registered under ClinicalTrials.gov NCT 044515664. https://clinicaltrials.gov/ct2/show/NCT04451564?recrs=ab&cond=fibromyalgia&cntry=CH&draw=2&rank=1 Registered on 3 July 2020. The trial was prospectively registered.
Protocol version {3}	The protocol number version 6, dated 30.03.2021.
Funding {4}	This study will financially be supported by the Swiss National Foundation, Grant Number 325130_182766. No other material or other support received.
Author details {5a}	Ledermann, K.,^1,2^ von Känel, R.,^1^ Berna, C.^3^, Sprott, H.^4^, Burckhardt, M. ^2^, Jenewein, J.^5^, Garland, E. L.^6^, Martin-Sölch, C.^2^ ^1^ University of Zurich, Department for Consultation-Liaison Psychiatry and Psychosomatic Medicine, Zurich, Switzerland ^2^ University of Fribourg, Department of Psychology, Chair of Clinical and Health Psychology, I-Reach Lab, Fribourg, Switzerland ^3^ Center for Integrative and Complementary Medicine, Division of Anesthesiology, Lausanne University Hospital. University of Lausanne, Switzerland. ^4^ University of Zurich, Arztpraxis Hottingen Zurich ^5^ Universitätsklinik für medizinische Psychologie und Psychotherapie, Medizinische Universität Graz, Austria. ^6^ University of Utah, College of Social Work, Center on Mindfulness and Integrative Health Intervention Development, Salt Lake City
Name and contact information for the trial sponsor {5b}	Prof. Dr. med. Roland von Känel University of Zurich, Department for Consultation-Liaison Psychiatry and Psychosomatic Medicine, Zurich, Switzerland Culmannstrasse 8 8091 Zürich, Switzerland Roland.vonkaenel@usz.ch
Role of sponsor {5c}	The Sponsor-Investigator is implementing and maintaining quality assurance and quality control systems with written standard operating procedures (SOPs) and working instructions to ensure that the trial is conducted and data are generated, documented, and reported in compliance with the protocol, good clinical practice (GCP), and applicable regulatory requirements. The Principal Investigators at all sites must have a manual of the relevant SOPs and WIs for the study on site and are responsible for proper training of all involved study personnel for the respective procedures*.* Monitoring and Audits will be conducted during the course of the study for quality assurance purposes.

## Introduction

### Background and rationale {6a}

Fibromyalgia (FM) is a chronic, painful, musculoskeletal disorder characterized by widespread pain, accompanied by a broad spectrum of associated somatic and psychological manifestations, including fatigue, sleep disturbances, stiffness, anxiety, and cognitive dysfunction [72]. It is one of the most prevalent chronic pain conditions [67] with an estimated prevalence between 0.5 and 4% with a ratio of 3.5% in women to 0.5% in men [47]. Like other chronic pain conditions, FM often leads to disability, affective disturbance, and poor quality of life and is also associated with high direct and indirect disease-related costs [58]. The etiology of FM is largely unknown. However, several factors appear to underlie the disorder, including dysfunction of the central nervous system (CNS) and autonomic nervous systems, neurotransmitters, hormones, immune system, and external stressors and psychological factors [6]. More generally, chronic pain is commonly associated with comorbid affective disorders (e.g., anxiety, depression) and cognitive deficits (e.g., memory impairment), suggesting on one hand critical involvement of higher order neural brain processing [13] and on the other the necessity to develop specific interventions targeting comorbid mental disorders, mood, and cognitive dysfunctions (see for instance [17]). Among the neural changes observed in chronic pain, there is increasing evidence for alterations in the dopaminergic (DA) system. Evidence supporting a hypodopaminergic state in chronic pain comes from both preclinical [56] and clinical data [46, 52]. Furthermore, accumulating evidence suggests that the mesolimbic DA system modulates the perception of nociceptive information and the affective symptoms of chronic pain [3]. Taken together, there are now multiple lines of evidence showing that chronic pain, including FM, leads to a hypodopaminergic state that results in enhanced pain sensitivity and might impair positively motivated behavior [64]. In addition, DA is involved in descending inhibitory modulation of pain transmission, which is an additional link between hypodopaminergia and chronic pain [59]. In a previous project of our group that aimed at investigating DA function in FM, we found a reduced DA function in FM patients and showed group differences in DA receptor binding in striatal regions between FM participants with and those without depression compared to healthy subjects [50]. In addition, we found a differential modulation of pain by DA in healthy controls and FM participants [50]. Findings from functional neuroimaging studies indicate that a network of brain regions, including the orbitofrontal cortex, the ventral (specifically the nucleus accumbens) and dorsal striatum, the amygdala, and the anterior cingulate gyrus form the so-called reward system [57]. In chronic pain, alterations in brain structural features, functional connectivity, or activity of these regions have been reported, compared to healthy controls, or people who tend to recover from acute pain ([3, 42, 74, 75]. The neural changes observed in regions associated with the brain reward system could provide a possible explanation for the high incidence of comorbid affective disorders in chronic pain patients [49]. In conclusion, the exact mechanisms by which the brain reward network modulates chronic pain have not been established yet. More specifically, although there is first evidence for a hypodopaminergic state in FM ([50, 74, 75], the effects of chronic pain on the ability to enjoy rewards and hence anhedonia have insufficiently been investigated. Finally, the exact role of DA in the modulation of chronic pain remains unclear. On another note, the current evidence-based guidelines for the treatment of patients with FM are inconsistent [66]. Recent meta-analyses conclude that optimal treatment interventions should include components aimed at enhancing adaptive cognitive and behavioral responses [1, 39], and broad improvements have been observed with treatment plans that include non-pharmacologic interventions [1]. This is in line with the current international guidelines that recommend aerobic exercise, cognitive-behavioral therapy (CBT), and multicomponent treatment as the first choice for the care of FM patients [66].

A growing body of research has demonstrated that mindfulness-based interventions are clinically effective for a wide range of conditions (for a review, see [40]) leading to them increasingly being used for the treatment of chronic pain conditions including FM ([60, 69]. A recent systematic review indicates that mindfulness-based interventions produce statistically significant, moderate effect size improvements in mood-related outcomes in FM [65]. However, most studies of mindfulness-based interventions have concentrated on negative affective-related constructs and have not given sufficient attention to the effects of mindfulness on positive affect and reward [27].

In contrast, Mindfulness-Oriented Recovery Enhancement (MORE) is a mind-body intervention specifically designed to enhance positive emotion regulation and natural reward processing by uniting complementary aspects of mindfulness training, CBT, and positive psychological principles into an integrative treatment strategy. MORE was originally designed as a behavioral medical intervention for addictive behaviors ([23, 28, 29], but was more recently adapted to address chronic pain among individuals receiving long-term opioid analgesic therapy. Two stage 2 randomized clinical trials (RCTs, *N* = 115 and *N* = 95) showed that MORE significantly reduces pain severity and pain-related functional interference [26, 31, 33–35], as well as opioid dosing [36], among chronic pain patients. In addition, the effects of MORE on reducing pain severity and opioid misuse were associated with increases in positive emotional processes, including positive affect, savoring, and meaning in life [24, 25, 30, 33–35]. In addition to these psychological effects, RCTs demonstrate that MORE is also associated with behavioral and neurophysiological changes in reward processing [26, 27, 31], including increases in the late positive potential of the EEG and enhanced corticostriatal activity during savoring of natural rewards [21, 33–35], suggesting that MORE might improve clinical outcomes by enhancing reward system function. It is however not clear whether or not the experimental modification of reward experiences represents a mechanism of change in MORE. Because our previous results showed dysfunctional DA responses to rewards in FM [49], the use of MORE in this group of patients could be very promising. Furthermore, no study so far has investigated the molecular underpinnings of MORE on the dopaminergic reward system.

In addition, daily measures of self-reported stress experiences and self-reported pain symptoms in FM indicate that stress exacerbates the pain feelings in everyday life of FM patients [20]. Furthermore, it has been hypothesized that FM patients may find it more difficult to mount a resilient affective response to stressful events if the force of negative affect (NA) compromises their resources of positive affect (PA) [76, 77]. In turn, PA is a source of resilience against pain and negative affectivity [76, 77], and momentary positive emotions, rather than more general satisfaction with life, are associated with increased psychological resilience [16]. In addition, in FM, deficits in PA regulation have been reported [76, 77]. Using ambulatory assessments (AA), MORE and mindfulness have been shown to significantly boost PA regulation and momentary positive emotions and enhance responsiveness to pleasant daily life activities [24, 25, 30, 33–35, 38]. The overall objective of this project is to investigate whether FM participants differ from healthy participants with regard to DA function, pain perception, neural responses to reward FM-related symptoms (including mood disturbances), and daily life affects, reward, stress, and pain experiences and with regard to emotion regulation and pain coping strategies before participating in the MORE intervention. Furthermore, we aim to investigate the effects of the MORE intervention for the first time in FM, and to measure its ability to restore DA function at a neurochemical level, to reduce pain, to enhance neural responses to reward; to reduce FM-related symptoms and to affect the experience of pain, stress, and reward in everyday life in FM patients; and to change emotion regulation and pain coping strategies. We will also investigate the short-term effects of MORE on FM-related symptoms and pain after 3 months. In an exploratory way, we will perform microbiota and genetic analyses to investigate emerging pathophysiological models of FM that could be associated to the DA dysfunction observed in FM as well the changes in mindfulness associated with MORE.

## Objectives {7}

### Aims and objectives

The objectives are as follows:

1. To compare the 18F-DOPA influx in striatal regions with 18F-DOPA PET before the MORE intervention between FM patients and a group of healthy controls and between FM patients that participated in MORE versus those assigned to a non-intervention control condition
2. To compare neural responses to reward with fMRI before the MORE intervention between FM patients and a group of healthy controls and between FM patients that participated in MORE versus those assigned to a non-intervention control condition
3. Explore changes in FM-related pain and mood symptoms after the MORE intervention
4. Explore changes in self-report measures of behavioral and biological stress in FM patients after the MORE intervention

Our hypotheses are:

1. We expect FMS patients to show a lower 18F-DOPA influx in particular in striatal regions than the healthy controls before (pre-test) the MORE intervention and the FM patients participating in 8 weekly sessions of MORE treatment to show an increased 18F-DOPA influx after the treatment (post-test) compared to a non-intervention FM control group.
2. We expect FM patients to show decreased neural responses to reward, in particular to striatal regions measured with fMRI than the healthy controls before (pre-test) the MORE intervention, and the FM patients participating in 8 weekly sessions of MORE treatment to show increased neural responses to reward measured with fMRI after the treatment (post-test) compared to a non-intervention FM control group.
3. We expect the FM patients participating in 8 weekly sessions of MORE treatment to show significant pain symptom reduction as evidenced by a significant reduction of VRS and BPI scores after the treatment (post-test) compared to a non-intervention FM control group.
4. We expect FM patients participating in the MORE intervention to show significant changes in mood and FM-related outcomes compared to a non-intervention FM control group.
5. We expect changes in everyday self-report measures of stress and pain levels including biological stress measures in FM patients versus healthy controls before the MORE intervention and between FM patients that participated in MORE versus those assigned to a non-intervention control condition.

## Trial design {8}

This is a multi-center interventional RCT with 3 time points: before the intervention (pre-test measures, T1), after completion of the intervention (post-test measures, T2), and 3 months after completion of the intervention (follow-up measures, T3). Participants in the non-intervention control condition will perform the T1 and T2 measures adjuvant 8 weeks apart. Study evaluation will be done by comparing within and between the groups. Healthy controls will perform T1 measures only. The potential effect will be assessed on a series of outcome measures (Table 1). Measures will take place at baseline T0, T1, T2, and T3.

**Table 1:** Questionnaires at assessment (T0), baseline (T1), and 3-month follow-up (T2)

Variables	T0	T1	T2	T3	
**Sociodemographic and medical variables**					
Demographics: for example, age, marital, status, education	x				
Medical history: for example, duration of FM, medication, treatment	x				
Lifestyle and health behavior: smoking history, alcohol consumption, exercise	x				
**Psychometric data**					
M.I.N.I International Neuropsychiatric Interview	x				
Beck Depression Inventory (BDI, [6])		x	x	x	
State-Trait Anxiety Inventory (STAI, [59])	x	x	x		
Profile of Mood States, POMS		x	x	x	
Quality of Life, WHOQOL [62]		x	x	x	
Fibromyalgia Questionnaire-Revised (FIQ-R) [9]		x	x	x	
Sleep quality, medical outcomes study sleep scale MOS (Stewart, 1988)			x	x	x
Five Facet Mindfulness Questionnaire [3]		x	x	x	
Pain Coping Questionnaire [12]		x	x	x	
Cognitive Emotion Regulation questionnaire (CERQ) [41]		x	x	x	
Savoring Beliefs Inventory [13]		x	x	x	
Temporal Experience of Pleasure Scale ([22])		x	x	x	
Edinburgh Handedness Inventory	x				
**Pain-related outcomes**					
Brief Pain Inventory (BPI, [15])		x	x	x	
Verbal Rating Scale for current Pain Intensity from SF-36 [70]		x	x	x	
Pain Disability Index [20]		x	x	x	

Before T1, we will perform the screening of the participants with regard to the inclusion and exclusion criteria (T0). Pre-test and post-test measures include each a 18F-DOPA PET scan at rest and a fMRI measure with the use of the reward task, the ambulatory assessment (AA) measures, the clinical and pain measures, and the fMRI measures. The follow-up consists in questionnaire assessments only, including the self-reported pain and clinical measures associated with PET and fMRI measures. The questionnaires will be presented through an online survey that is suitable for RCTs (Research Electronic Data Capture, RedCAP) and available at the Universities Fribourg and that can be filled at home.

**Fig. 1:**
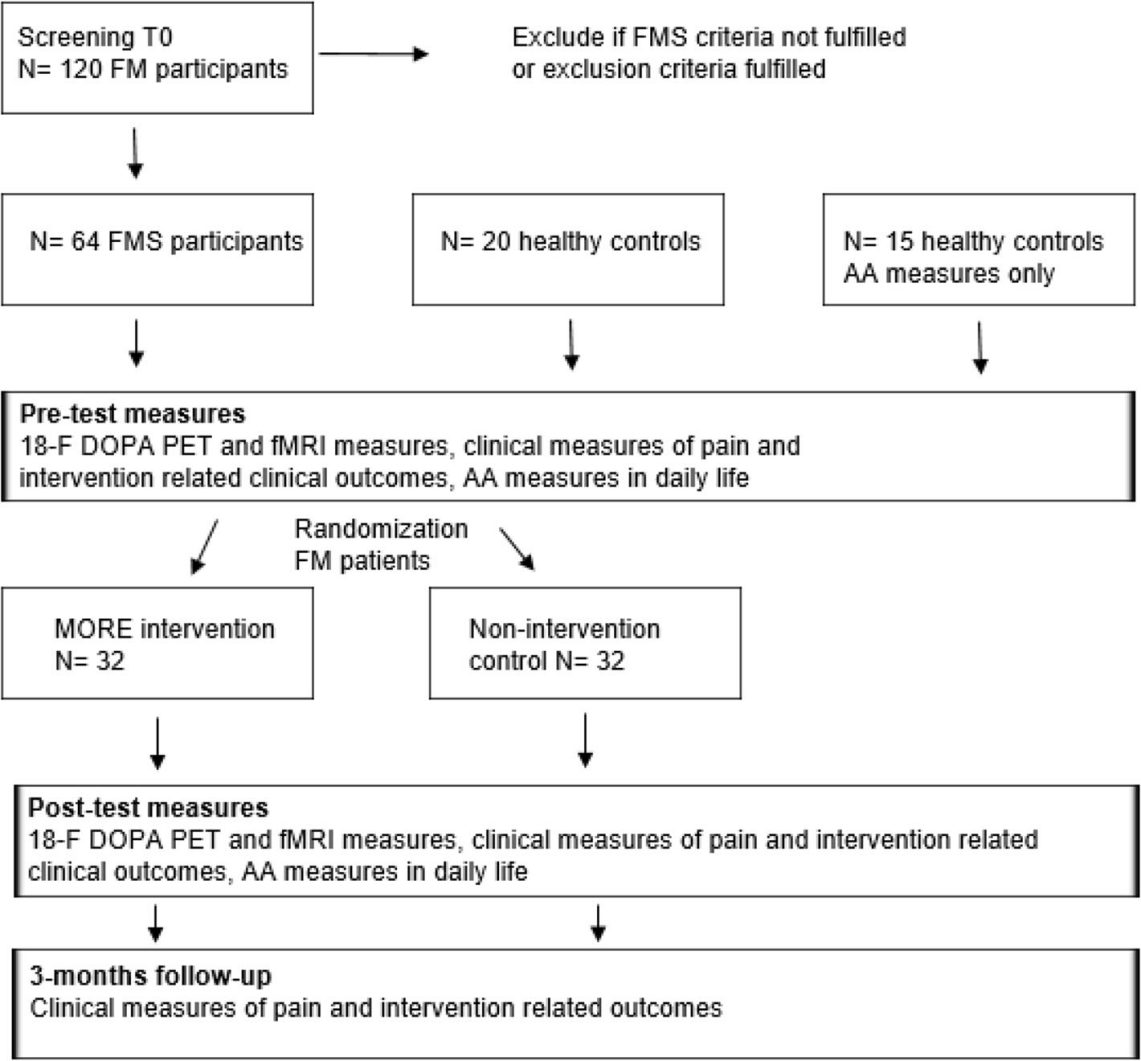
Flowchart of the intervention study

The non-intervention FM control will undergo the same measures at pre-test and post-test, with a post-test planned on average after 8 weeks. They will not participate in any specific intervention, but will be given the opportunity to participate in MORE after the follow-up assessment (Fig. 1).

## Methods: participants, interventions, and outcomes

### Study setting {9}

The study is a multi-center study with 3 centers and includes the University Fribourg, Department of Psychology; the University Hospital Lausanne (CHUV), Center for Integrative and Complementary Medicine & Pain Center; and University Hospital Zurich, Department of Consultation-Liaison Psychiatry and Psychosomatic Medicine. All 3 centers are based in accredited Swiss Universities, two of them are located in University Hospitals. The MORE intervention will be given in each of these centers in order to access a larger pool of participants. The PET scans will be performed at the PET-Center of the Department of Nuclear Medicine at the University Hospital Zurich. The fMRI measures will be performed at the Department for Neuroradiology at the University Hospital Zurich. Data collection takes place in Switzerland only.

### Eligibility criteria {10}

The following groups of participants will be included: (a) 64 female subjects fulfilling the classification criteria of the American College of Rheumatology for FM (ACR 2011 criteria) [73] and without any psychiatric condition according to the ICD-10 and (b) 20 healthy women without a history of chronic pain or any mental disorder for the PET measures, and a total of 15 healthy women for the daily ambulatory assessments. The groups will be matched on age. All participants will be right-handed and older than 18 years old. Subjects will be excluded if they are pregnant; if they have a history of neurological disorders, current substance or tobacco abuse, current and past substance dependence, schizophrenia spectrum disorder, and any other form of chronic pain (except FM for the FM group); and if they have been treated with a medication affecting the central DA system in the 3 months preceding the scanning session (including opioids, neuroleptics, antileptics, antidepressants, and lithium). Participating in another RCT or some form of individual or group psychotherapy focusing on pain management is prohibited.

### Who will take informed consent? {26a}

Informed consent will be obtained by trained and authorized study personal. The investigator will explain to each participant the nature of the study, its purpose, the procedures involved, the expected duration, the potential risks and benefits, and any discomfort it may entail. Each participant will be informed that the participation in this study is voluntary and that she may withdraw from the study at any time and that withdrawal of consent will not affect her subsequent medical treatment. All participants will be provided with a participant information sheet and a consent form describing the study and providing sufficient information for participants to make an informed decision about their participation in the study.

### Additional consent provisions for collection and use of participant data and biological specimens {26b}

Consent for each participant will be obtained that in case of withdrawal, the biological materials and health-related data collected before withdrawal will be stored and analyzed in coded form and used in the analyses. Anonymization of the biological material and personal data may be dispensed with if the person concerned expressly renounces this right when revoking consent.

## Interventions

### Explanation for the choice of comparators {6b}

We have the following reasons to justify the use of a design with a non-intervention control group and not an active control group. Placebo group interventions without specific effects can elicit so-called resentful demoralization [61], leading to increased drop-out risk in this group. With regard to the study of [26, 31] indicating more than 20% drop-out during the treatment, it is important to minimize this risk. Providing a non-efficacious treatment to patients having a long history of mostly not efficacious treatment behind them as it is the case for FM patients is questionable. Because of their symptomatology, it is difficult for them to commit to a treatment and they often encounter mobility difficulty due to pain symptoms, so it would be a waste of time and energy for them to come to our centers for an intervention without any specific effects. From a methodological point of view, it is difficult to provide comparable support group interventions among different groups as the interaction between the participants and the reaction of the therapist to them cannot be fully controlled. This is particularly complicated when the intervention is given in 3 different centers and might impair the validity of the intervention. For these reasons, RCTs in patients with somatic diseases use in the great majority non-intervention designs to assess the effects of psychological interventions.

### Intervention description {11a}

FM participants will participate in the MORE intervention or in a non-intervention control group. The non-intervention control group does not include any intervention. The MORE treatment is manual-based [28], consists of 8 sessions, and offers instruction in applying mindfulness and related skills to the following topics: discriminating between nociception, pain, and suffering; gaining awareness of automaticity and coping habits in chronic pain; disrupting the link between negative emotions, catastrophizing, and pain experience through reappraisal; refocusing attention from pain and life stressors to savor pleasant experiences; promoting acceptance instead of suppression of experience; and developing a mindful recovery plan. Mindfulness training will involve mindful breathing and body scan techniques, with an emphasis on developing metacognitive awareness and shifting attention from affective to sensory processing of pain and craving sensations. Sessions will be audio recorded for control of therapists’ adherence with the manual. MORE participants will be asked to engage in daily 15-min mindfulness practice sessions at home guided by a MP3 recording of the meditations. MORE is a manualized intervention that is centered on three therapeutic processes: mindfulness, reappraisal, and savoring. The MORE manual and the meditations are translated in French and in German. The non-intervention control group includes continuation of treatment as usual.

### Criteria for discontinuing or modifying allocated interventions {11b}

Discontinuing or modifying allocated interventions is not possible.

### Strategies to improve adherence to interventions {11c}

The sponsor-investigator is responsible to have written SOPs and WIs in place for the study and to provide those to all participating study sites. The principal investigators at all sites must have a manual of the relevant SOPs and WIs for the study on site and are responsible for proper training of all involved study personnel for the respective procedures*.* Adherence to intervention protocols will be controlled by recording the MORE sessions. EG, the founder of MORE, or KL will listen to the sessions to control for therapists’ adherence. To assess compliance with the intervention, we ask participants to report the mean time practice resp. meditation time per day, each week when they come for the MORE course. We will record the number of MORE courses completed for each participant and allow up to missing 2 MORE courses.

### Relevant concomitant care permitted or prohibited during the trial {11d}

FM participants will be withdrawn from the study if they receive treatment with medication affecting the central DA system, including opioids, neuroleptics, antileptics, antidepressive, and lithium, or if they have to undergo an examination with radiopharmaceutical or radioactive marked substances or if they begin another regular psychotherapeutic treatment or participate in another clinical trial during the course of the study until completion of the post-test measures. All other treatments are allowed during the course of the study. For healthy participants participating in PET measures at pre-test, the same conditions are applicable until the end of pre-test measures. For healthy participants not participating in the PET measures, the same conditions are applicable with exception of the examination with radiopharmaceutical or radioactive marked substance. Concomitant treatment will be recorded at each study visit in the eCRF.

### Provisions for post-trial care {30}

Not foreseen.

### Outcomes {12}

To minimize bias, we will use only standardized questionnaires to assess the primary outcomes as recommended by Outcome Measures in Rheumatology Clinical Trials (OMERACT) [10].

#### Main primary outcome

1) A.18F-DOPA influx: the difference between FM patients and healthy controls at pre-test and between pre-test and post-test in FM

1) B. Measures of pain symptoms: the difference between FM patients and healthy controls at pre-test and between pre-test and post-test in FM in the scores of the 1-item Verbal Rating Scale (VRS) [70] and of the Brief Pain Inventory (BPI) [15].

#### Main secondary outcomes

2) The percent BOLD signal change in striatal activity during the reward task: the difference between FM patients and healthy controls at pre-test and between pre-test and post-test in FM patients
3) The correlation between daily practice of mindful breathing and savoring (minutes) and increased striatal activity and 18F-DOPA influx
4) Changes in pain-related and other clinical outcomes: the difference between FM patients-healthy controls at pre-test and between pre-test and post-test in FM on scores of quality of life (WHOQOL Brief [62];) and mood disturbances (State-Trait Anxiety Inventory (STAI [48];), Beck Depression Inventory (BDI)-II [43], Fibromyalgia Impact Questionnaire (FIQ [7];), Pittsburgh Sleep Quality Index (PSQI; [14]), and Emotion Regulation Questionnaire (CERQ; [51])
5) Effects of the MORE intervention on self-reported pain, positive affect, and stress in daily life measured with AA: the difference between FM patients and healthy controls at pre-test and between pre-test and post-test in FM patients

### Participant timeline {13}

The overall study duration is 4 years from June 2021 to June 2025. Recruitment will take place during the entire study duration as we will perform 4 successive groups of the MORE intervention. The study duration per participant will be between 6 and 8 months, depending on scanner availability for the pre- and post-test measures. As soon as we have a minimum of 10 FM participants (minimum 5 participants and maximum 10 participants per MORE group), we will start with the first MORE group. The first measures will ideally be performed on healthy participants. The FM participants will be randomized after screening, and the ones assigned to the MORE intervention will undergo the pre-test measures of FM first. We plan 4 to 5 weeks to perform the pre-test measures for the participants allocated to the MORE group (2 participants per week). The first MORE will take place in the center where we will have first recruited the necessary number of participants. During the duration of the first MORE group (8 weeks), we will make the pre-test measures for the non-intervention control group and begin the pre-test measures for the second MORE group. This will allow us to begin with the second group as quickly as possible after the completion of the first group. During the second MORE group, we will perform the post-test measures for the first MORE group participants and test the non-intervention controls of the second MORE group. During fall 2021, we will test participants of the healthy control group and recruit the participants for the next two MORE groups that will take place in winter/spring 2022, according to a similar schedule for the first 2 groups. In parallel, we will perform the follow-up measures for MORE 1 and MORE 2. FM participants allocated to the non-intervention group will have the possibility to participate in a MORE group at the earliest after all participants of the group have completed follow-up measures, i.e., around 20 weeks after pre-test-measures (8 weeks delay for post-test measures + 12 weeks after post-test measures). In the years 2023 and 2024, we will complete the measures for the control group as well as the follow-up measures for the FM groups.

### Sample size {14}

Sample size justification:

The power analysis for the primary outcome is based on the following results:

• A study comparing 18F-DOPA influx between FM participants (*N* = 6) and a healthy control (*N* = 8) group [74, 75] yielded a mean effect size of 1.17; using this effect size, a power analysis with G*Power [19] showed that a group of 12 participants is optimal to obtain significant results with *T*-test comparing 18F-DOPA uptake between FM and healthy participants at baseline. We would round up to 20 to accommodate for technological problems, as the first measures will be done in healthy participants.

• Garland et al. [32] found significant neurophysiological changes after MORE treatment with EEG measure in 29 participants (F1,26 = 4.47; ηpartial^2^ = .15) in response to reward before and after the MORE intervention in chronic pain patients with sample sizes of *N* = 11 (active group) and *N* = 18 (support group). Using this effect size, a power analysis with G*Power [19] showed that 14 participants are necessary for an ANOVA with 2 factors and repeated measures to be significant. However, results obtained with EEG cannot be directly compared to PET measures, and there are so far no available studies with FDOPA in that context.

The power analysis for the secondary outcomes is based on the following results:

• A randomized controlled study of the MORE intervention by Garland et al. [26, 31] evidenced significant reductions in self-reported pain severity with medium effect size (Cohen’s *d* = 0.5, effect size *F* = 0.46) after the MORE intervention in a starting group of 115 participants and of 69 at post-intervention measures (*N* = 31 for MORE and *N* = 38 for support group). Based on these estimates, an ANOVA to be significant should have a minimal sample of *N* = 20 participants after treatment in each group.

• A randomized controlled study by Garland et al. [33–35] found significant effects of MORE on self-reported pain, stress, and positive affects in daily life (with AA measures) in a group of *N* = 30 using multilevel analyses, with effect sizes yielding to a minimal sample of 20.

• In their randomized clinical trial of the MORE intervention in chronic pain patients with opioid abuse, [26, 31] found a 20% drop-out between the beginning and completion of the intervention.

• In order to have a sample size sufficient to obtain significant results for the primary outcomes (FDOPA) and clinical pain measures as well as for the secondary outcomes (in particular daily life measures), we consider a minimal sample of *N* = 20 in each FMS group. Considering a 20% drop-out in total yields to a starting sample of 25 participants in each FMS group (MORE and non-intervention control). To account for data loss related to technical problems as well as for the average size of the MORE group of *N* = 8, we will round up to *N* = 32 in each FMS group.

### Recruitment {15}

Patients will be recruited from the interdisciplinary outpatient clinic for pain at the University Hospital Zurich, from the Department of Consultation-Liaison Psychiatry and Psychosomatic Medicine at the University Hospital Zurich, from the Neurology Department and interdisciplinary outpatient clinic for pain at the Canton Hospital Fribourg, from the outpatient pain clinic at the Division of Anesthesiology as well as the service of rheumatology at the Lausanne University Hospital, from private practices, and from adds on websites of the Rheumaliga, as well as patient groups such as the Fibromyalgieforum Schweiz in the German-speaking and French-speaking parts of Switzerland. The healthy controls will be recruited through ads, word of mouth, and from our previous and current studies as well. When interested participants call into one of the study telephone lines, they will be told about the study and screened for eligibility using predetermined scripts. If potentially eligible, an in-person visit (90-min duration) at the study center will be scheduled. This first visit can be the start of the study, or this start can be reported, depending on the clinical situation. At the study start, eligibility will be confirmed, consent provided, and the initial assessment completed.

## Assignment of interventions: allocation

### Sequence generation {16a}

We will use a simple randomization procedure with a blocked size of 5, 6, or 8 according to the recruitment flow. Randomization will be performed with an online program (sealed envelope, https://www.sealedenvelope.com) with the following randomization scheme: FMS MORE/FMS non-treatment control. The randomization will be performed for each group independently, before the pre-test measures by the data manager. Randomization tables will be deposited on RedCAP. There is no randomization of the healthy control groups for allocation in the PET or AA group.

### Concealment mechanism {16b}

The list is concealed by instructed office personnel in the study center of the University Fribourg, Switzerland, who is unaware of any patient information. The randomized participants will join the MORE group or non-intervention control.

### Implementation {16c}

The 64 FM participants will be assigned to the MORE treatment or to a non-intervention control condition in a randomized way. As the study progresses, participants coming off the waitlists will also be added to the ongoing groups. We will begin with one MORE group running in Zurich and in Lausanne, but will add additional groups as numbers require. For the healthy participants, we will fill first the PET group *N* = 20 and allocate the rest to the AA group.

## Assignment of interventions: blinding

### Who will be blinded {17a}

Because there is no active control group or no placebo intervention in this study, the study staff and the participants will be unblinded.

### Procedure for unblinding if needed {17b}

N.A.

## Data collection and management

### Plans for assessment and collection of outcomes {18a}

A detailed overview of questionnaires and measures is given in Table 1. The schedule of enrolment, interventions, and assessments is provided in Table 2. Data management including data entry, coding, security, and storage will be provided by the Web-based application Research Electronic Data Capture **(**REDCap) 10.5195/jmla.2018.327 that is available at the University of Fribourg. It is Health Insurance Portability and Accountability Act (HIPAA)-compliant and highly secure.

**Table 2:** Schedule of enrollment, interventions, and assessments

	From June 2021	> 1 day	> 2 days	After 8 weeks MORE intervention/waitlist	
Timepoint**	***-t***_***1***_	***t***^**0**^	***t***_***1***_	***T***_***2***_	***T***_***3***_
**Enrolment**	**Information screening**		**Pre-test measures**	**Post-test measurements**	**3-month follow-up**
**Eligibility screen**	**X**				
**Informed consent**	**X**				
**Baseline data**	**X**				
**Allocation**		**X**			
**Interventions**					
***F-DOPA PET scan***			**x**	**X**	
***MR scan***			**X**	**X**	
***Reward task***			**X**	**X**	
**Assessments**					
***Medical history*** ***Clinical psychiatric interview*** ***Physical examination, tender point evaluation*** ***Self-report scales***	**X**				
***Intervention related clinical outcomes***			**X**	**X**	**X**
***AA sampling***			**X**	**X**	

### Sociodemographic data

Data to be collected at baseline include age, sex, marital status, education level, and professional status. Measures of functioning include a measure of quality of life with the World Health Organization quality of life questionnaire WHOQOL Brief questionnaire [62] and the Fibromyalgia Impact Questionnaire-Revised (FIQ-R [7];) that also includes a measure of fatigue. Measures of sleep quality include the medical outcomes study sleep scale (MOS [63]; [44]) that has been widely used in rheumatology research and is recommended by OMERACT-10.

### Questionnaires

#### Pain-related outcomes

• *Pain severity and functional interference*: The Brief Pain Inventory (BPI) is used to obtain information on self-reported measures of pain severity and functional interference using the BPI [15]. Pain magnitude is queried by four items that ask about pain now, worst pain, least pain, and average pain. Items use numeric rating scales anchored by 0 (no pain) to 10 (most severe pain). Pain interference consists of seven items that ask about how pain interferes with aspects of daily living using numeric rating scales anchored by 0 (no interference) to 10 (completely interferes).

• *Current pain intensity*: The 1-item Verbal Rating Scale (VRS) from the SF-36 [70] is used to measure current pain intensity. The VRS has proven itself over decades as a valid, reliable, and change-sensitive measure of subjective pain [70].

• *Pain interference with functional impairment*: To measure the degree to which pain interferes with function in major life areas, we will use the 7-item Pain Disability Index (PDI) [18].

#### Other clinical measures

• *Severity of depressive symptoms*: To assess the severity of depressive symptoms, the Beck Depression Inventory (BDI)-II ([4, 43], a self-report questionnaire, will be used.

• *State and trait anxiety*: The State-Trait Anxiety Inventory (STAI) will be used as a measure of state and trait anxiety [48].

• *Mood states*: The Profile of Mood States (POMS) [53] will be used to assess transient, distinct mood states. The POMS measures six different dimensions of mood swings over a period of time with high sensitivity to change.

• *Quality of life*: To measure the quality of life, we will use the quality of life scale from the World Health Organization WHOQOL Brief questionnaire consisting of 26 items [62]. The WHOQOL Brief has good to excellent psychometric properties of reliability and performs well in preliminary tests of validity [68].

• *Function, impact, and overall symptoms of fibromyalgia*: The revised Fibromyalgia Impact Questionnaire (FIQ-R) [7] is a commonly used and validated 9-item instrument in the evaluation of fibromyalgia (FM) patients.

• *Sleep quality* will be assessed using the sleep quality, medical outcomes study sleep scale (MOS) [44, 63] that includes 12 items assessing sleep disturbance, sleep adequacy, somnolence, quantity of sleep, snoring, and awakening short of breath or with a headache.

• *Mechanisms underlying the MORE intervention*: For mechanisms related to the MORE intervention, we will assess with the following questionnaires: nonreactivity: Five Facet Mindfulness Questionnaire [2], the reinterpretation of pain sensations (Subscale of Coping Strategies Questionnaire, [11]), positive reappraisal (Subscale of Cognitive Emotion Regulation Questionnaire CERQ) [37], and savoring (Savoring Beliefs Inventory) [12], according to [26, 31]. They aim to understand the mechanisms related to the MORE intervention.

#### PET data acquisition

PET images will be acquired at the Department of Nuclear Medicine at the University Hospital Zurich. MRI overlay images will be acquired at 3 Tesla at the Department of Nuclear Medicine at the University Hospital Zurich, using T1-weighted sequence (MP-RAGE) to provide an anatomical framework for image analysis. 18F-DOPA is a well-validated measure of presynaptic DA function [45], and previous studies showed differences in 18F-DOPA binding between FM and healthy participants at rest [74, 75]. Subjects will have an intravenous catheter placed in their forearms. One hour before scanning, they will receive as routinely an oral dose of carbidopa 100 mg, a peripheral DOPA decarboxylase blocker and entacopone 400 mg, a peripheral catechol O-methyltransferase antagonist, and an additional 50 mg dose of carbidopa 30 min before 18F-DOPA injection in order to provide increased availability of 18F-DOPA for striatal uptake. The purpose of the premedication regimen is to limit the metabolism of the 18F-DOPA tracer by peripheral enzymes, i.e., DOPA decarboxylase and catechol O-methyltransferase, thereby maximizing central uptake [54]. Subjects will lay quietly before administration of the tracer to allow them to habituate into the environment and to relax. Subjects will then be injected with approximately 100 MBq of 18F-DOPA. Dynamic scanning will be performed up to 100 min after injection of tracer, but at least between 40 and 90 min after injection (cite https://www.ncbi.nlm.nih.gov/pmc/articles/PMC5584692/). Scans will be acquired on a PET/CT Discovery 690 scanner. Subjects will undergo the PET and MRI measures on the same day at the same place. Participants will have time to rest and to have lunch between both measures. PET measures will take place at pre-test and post-test. Pre-test measures take place 6 weeks to 1 day before the beginning of the MORE intervention. Post-test measures take place from 1 day after completion of the MORE intervention (i.e., + 8 weeks and 1 day after the beginning of the MORE intervention) to 6 weeks after completion of the MORE intervention (i.e., + 14 weeks after the beginning of the MORE intervention).

#### Neural activation in response to reward measured with fMRI

The fMRI data acquisition will take place at the Department of Nuclear Medicine at the University Hospital Zurich. To measure brain structure and function, we use a 3.0-Tesla whole-body scanner. The measures include T1-weighted images (structural MRI)

- Resting state functional MRI
  Task free: Subjects are instructed to just lie quietly in the scanner and to think of nothing in particular and let their mind wander.
- Task-based functional MRI during the reward task
  Total time in the scanner will be about 45 min.

#### Reward task (wheel of fortune task) (adapted from [5])

**Fig. 2:**
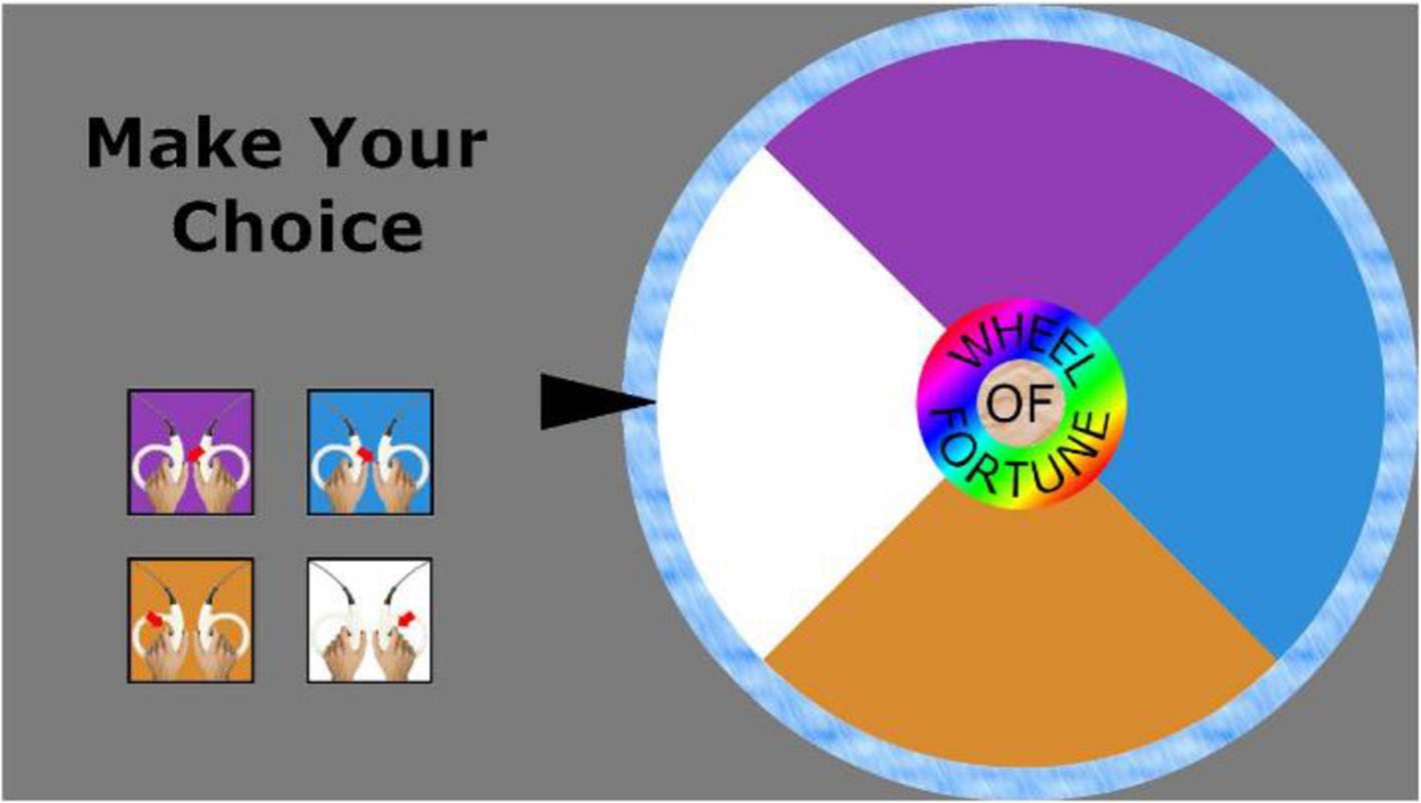
The reward task wheel of fortune

The subjects will perform a reward task adapted from a version of a wheel of fortune task [5], to measure reward during fMRI. The task consists in a computer screen showing a wheel of fortune presented to the participants (see Fig. 2). The wheel has four sections of the same size but with four different colors. The task is composed of two types of trials. There are rewarded and neutral trials. In the first ones, participants can choose between the four colors of the wheel and then press the corresponding button, and they will receive a large or a small monetary reward on a pseudo-randomized schedule every third time in average. In the second type of trials, participants can choose between the four colors of the wheel and then press the corresponding button, but receive no feedback on their answer. In both conditions, after pressing the button, the wheel starts spinning and then stops and the cursor indicates a color. In the reward condition, there are two possibilities: if the cursor indicates the color chosen by the participant, the participant wins some money; if it does not stop on the selected color, the participant wins no money. For the “neutral” condition, the cursor shows any color, but there is no feedback. The participants are informed about the different types of trials with the presentation of a cue information at the beginning of the trial. The subjects are instructed that they will receive the sum shown at the end of the experiment. The subjects will be asked to rate their mood with a visual analog scale in regular intervals. A short training of the task will take place before the beginning of the scanning session outside and inside the scanner. The outcome is the difference in neural activation before and after the 8-week MORE intervention. We will compare neural activation related to reward at pre-test and at post-test between the FMS participants of the MORE group and the participants of the waitlist.

#### Ambulatory assessment measures and physiological measures

To investigate the effects of the MORE intervention on daily affects, stress, and reward experiences, we will use ambulatory assessment (AA) measures. Primary outcomes related to the AA measures include (1) self-reported measures of pain; (2) self-reported measures and physiological measures (CAR, alpha-amylase awakening, and daily profile of cortisol and alpha-amylase) of stress, (3) measure of positive affect, and (4) measures of reward experience. They will be compared between FM participants and healthy controls before the MORE intervention and before and after the MORE intervention between both FM groups. AA self-reports of pain, stress, and reward and positive experiences in daily life will be performed using an adaption of the Experience Sampling method [71] to assess the stress and reward experience in the daily living environment. The items related to pain are defined according to a pain diary that we have developed, tested, and validated in 50 chronic pain patients at the University of Fribourg. The self-reported items measuring stress, positive and negative affects, and reward experiences have additionally been adapted and validated in the framework of another research project [41]. All self-assessments are rated on a 7-point Likert scale. The participants will receive an iPod and perform self-assessments during 5 weeks five times a day, during the week preceding or following the PET measures before and after the MORE intervention. At the end of each day, participants randomly assigned to MORE will record the number of minutes spent engaged in the mindfulness, reappraisal, and savoring practices taught in the MORE intervention to provide a measure of adherence with the home practice associated with the MORE training. Biological measures of stress in everyday life will be assessed by collecting saliva samples 6 times a day (2 for cortisol awakening response (CAR) and for the salivary alpha-amylase (AA) awakening, 4 for daily profile) for a total of 3 days. Saliva samples will be obtained by using the passive drooling method (Salicap, IBL International Hamburg, Germany). Saliva samples will be stored overnight in the participant’s refrigerator. The saliva samples will be then sent to the Department of Psychology at the University Fribourg, where they will be stored at −20 °C in a freezer dedicated to research with restricted access. The CAR resp. AA awakening profile and the daily profiles will be used as biological indexes of stress reactivity in everyday life [71]. The outcome is the difference between pre-test and post-test and the comparison between the MORE group and the waitlist group. All samples will be sent for analyses at the end of the study.

#### Follow-up measures

Follow-up measures include all questionnaires presented online via RedCAP, 3 months after the end of MORE intervention (+ 20 to + 24 weeks after MORE beginning for the non-intervention control group).

### Plans to promote participant retention and complete follow-up {18b}

Participation in this study is voluntary. If a participant wishes to retain from participating, she can stop the study without justification. In case of withdrawal, the biological materials and health-related data collected before withdrawal will be stored and analyzed in coded form and used in the analyses.

### Data management {19}

All study data must be archived for a minimum of 10 years after study termination or premature termination of the clinical trial. Study data will be archived at the University Fribourg.

For data recording, we will use electronic case report forms (eCRF) from REDCap which are available at the University Hospital Zurich, University Fribourg, and at the CHUV. For each participant, an individual CRF is maintained. The CRF is coded for each participant with protocol identifier plus randomization number based on the guidelines for an acceptable coding of trial subjects available on www.swissethics.ch. Source data in this study is all information in original records, certified copies of original records of clinical findings, questionnaires, observations, or other recorded activities collected during the study only. All collected data during the study will be transferred to the participant’s CRF. Direct access to source documents will be permitted for purposes of monitoring, audits, or inspections. Access to the project plan, dataset, and statistical code, during and after the research project (publication, dissemination), will be determined by the PI. Coding will be done using participant numbers and participants will be given a number in a chronological order with the first number differentiating between healthy controls and FM participants in the screening log. Data exchange between the centers will be done using coded data. For interim and for the main analyses, the (coded) data of the study centers will be merged into one file to perform the analyses.

### Confidentiality {27}

The investigators are liable to treat the entire information related to the study and the compiled data strictly confidential. Any passing-on of information to persons that are not directly involved in the study must be approved by the owner of the information. Direct access to source documents will be permitted for purposes of monitoring, audits, and inspections. Data generation, transmission, archiving, and analysis of personal data within this study strictly follow the current Swiss legal requirements for data protection. Prerequisite is the voluntary approval of the participant given by signing the informed consent prior start of participation of the clinical trial. Individual participant medical information obtained as a result of this study is considered confidential and disclosure to third parties is prohibited. Participant’s confidentiality will be further ensured by utilizing participant identification code numbers to correspond to treatment data in the computer files. Such medical information may be given to the participant’s personal physician or to other appropriate medical personnel responsible for the participant’s welfare, if the patient has given his/her written consent to do so. Data generated as a result of this study are to be available for inspection on request by the monitors and by the Cantonal Ethics Committee.

### Plans for collection, laboratory evaluation, and storage of biological specimens for genetic or molecular analysis in this trial/future use {33}

Biological specimens obtained under this protocol will be stored in coded form (protocol identifier plus randomization number), in freezers located in an access-controlled room at the University Fribourg. The cooling of the freezer system is guaranteed as an increase in temperature will lead to an alarm that goes directly to the PI’s designee’s phone or any other predetermined person in charge for the cooling system. When coded data is shared, the key to the code will not be provided to collaborators, but will remain in the hands of the PI and her designees. Saliva samples will be collected in coded form and sent to the Department of Psychology at the University Fribourg with a prepared envelope for storage. Coded saliva samples for cortisol and amylase analyses will be frozen and stored at −20 °C in a freezer located in the access-controlled room at the University Fribourg. After collection of saliva samples from all participants, the samples will be shipped to the Dresden Lab Service located at the University of Dresden, an institution operating under the Declaration of Helsinki.

Saliva samples will be destroyed from the laboratory after the analyses. If a participant withdraws from the study, all collected data will be kept and used for data analyses.

## Statistical methods

### Statistical methods for primary and secondary outcomes {20a}

#### Planned analyses

In general, the main analyses will follow a 2-factorial design treatment group (FM MORE intervention group and FM non-intervention control) × time (pre-test and post-test) for the main analyses comparing the pre-test and post-test measures in the FM participants having participated in the MORE intervention versus the FM participants of the non-intervention control. These analyses will be done with ANOVA’s for the questionnaires, pain threshold measures, and CAR measures as well as for extracted 18F-DOPA influx estimate parameters and beta values for the fMRI analyses. AA self-report measures will be computed as means and analyzed with ANOVA’s. For analyses related to follow-up, we will include a third level (follow-up) to the time factor.

#### Primary analyses

##### Main outcome 18F-DOPA

State-of-the-art image processing will be used to analyze PET and fMRI images. PET image pre-processing and analysis will be done using PMOD (PMOD software, PMOD technologies Zurich) and SPM (Wellcome Department of Imaging Neuroscience, London, UK) for the 18F-DOPA study. The most often used method to quantify biochemical function from 18F-DOPA PET image is the multiple time graphical approach (MTGA) that provides rate constants (Ki) for the storage of 18F-DOPA within regions of interest (ROIs) placed over the striatum [8] and in regions associated with the processing of reward. Images from each dynamic DOPA PET dataset will be aligned and parametric images of 18F-DOPA influx (Ki) will be created for each subject. The Ki images will be transformed into standard stereotactic space. Regional Ki values of striatal regions (putamen, caudate, nucleus accumbens) will be analyzed with statistical parametric mapping for comparison of regional Ki values on a voxel by voxel basis. Group comparisons will be tested using independent samples *T*-test for pre-test measures; treatment group × time comparisons will be analyzed with two-factorial ANOVA’s including Ki values obtained in the nucleus accumbens and the caudate to test the primary outcomes. Regional Ki values can be reported into SPSS for the main analyses and for associations with the other data. Exploratory analyses can include additional moderation and mediation analyses on the basis of the results.

##### Main outcome: questionnaire measuring pain: VRS and BPI scores

The analyses for the VRS and BPI scores will be performed with a two-factorial (treatment group × time) ANOVA. In case of not normally distributed data, we will use a transformation in Z-scores, rather than using non-parametric tests. Pre-test comparisons with healthy controls will be performed with *T*-tests.

#### Secondary analyses

##### Secondary outcome: neural responses to reward

The reward task will be performed in the fMRI scanner, and we will correlate the 18F-DOPA influx with the striatal activation obtained with the fMRI task. In order to measure the behavioral differences in the fMRI task results, in the ambulatory assessment data, and in the intervention program’s efficacy between the groups tested, we plan to use different kinds of variance analyses and multilevel analysis. The number of participants for each part of the study has been determined in order to assure a good statistical power according with this specific research domain (e.g., including potential dropout, outliers). fMRI data analysis will be performed with state-of-the art software freely available online. T1-weighted scans will be analyzed with regard to cortical thickness and subcortical volumes using surface-based morphometry implemented in FreeSurfer software suite 5.0.1 (http://surfer.nmr.mgh.harvard.edu). This includes a fully automated method. Briefly, this processing includes (1) motion correction, (2) removal of non-brain tissue, (3) automated Talairach transformation, (4) segmentation of the subcortical white matter and deep gray matter volumetric structures (amygdala, hippocampus, thalamus, caudate, putamen, pallidum, nucleus accumbens, ventricles), (5) intensity normalization, (6) tessellation of the gray matter/white matter boundary, (7) automated topology correction, and (8) surface deformation following intensity gradients to optimally place the gray/white and gray/cerebrospinal fluid borders at the location where the greatest shift in intensity defines the transition to the other tissue class. Freesurfer morphometric procedures have been demonstrated to show good test-retest reliability across scanner manufacturers and across field strengths. Obtained subcortical volumes will be normalized by individual intra-cranial volumes for further statistical analysis. Exploratory approaches will involve a vertex-based analysis across the whole brain. Diffusion MRI scans will be analyzed for structural connectivity with FMRIB software library 4.1.9 (FSL, http://fmrib.ox.ac.uk/fsl). Functional MRI scans will be analyzed for activity and functional connectivity with the latest version of Statistical Parametric Mapping (SPM, http://www.fil.ion.ucl.ac.uk/spm). Functional MRI data will be pre-processed according to the following steps: (1) slice timing correction, (2) realignment, (3) linear and non-linear normalization onto a standard EPI template, (4) voxel re-sampling to 2×2×2 mm^3^, (5) smoothing with a Gaussian kernel of 6 mm full width at half maximum, (6) detrending, (7) filtering (such that frequencies 0.01 < *f* < 0.08 Hz passed the filter), and (8) regressing out the variance of nuisance covariates. With REST toolbox 1.6, for each subject and each specified region of interest (atlas-based specification), mean signal time courses will be extracted and cross-correlated. Next, correlations will be *r*-to-*z* transformed for group-level statistics. These *z*-values can be used for comparisons across (cross-sectional) and within (longitudinal) groups or for parametric correlations with psychometric measures. A region-of-interest approach will be used in subcortical regions involved in reward to obtain beta-weights (parameter estimates) that can be correlated with the 18-FDOPA estimate parameters as well as with AA self-report means and other outcomes.

##### Secondary outcome: clinical and FM pain-related symptoms

The analyses of the measures related to the clinical and FM pain-related symptoms including measures of pain intensity and severity, functioning, sleep, mood, and quality of life will be analyzed with a two-factorial (treatment group × time) ANOVA. In case of not normally distributed data, we will use a transformation in *Z*-scores, rather than using non-parametric tests. Pre-test comparisons with healthy controls will be performed with *T-*tests. For the analyses of the effects at follow-up, we will add an additional level to the time factor (pre-test, post-test, and follow-up). Exploratory analyses can include additional moderation and mediation analyses on the basis of the results.

##### Secondary outcome: daily affect, reward, stress, and pain experience

The AA measures yield intensive longitudinal data that are clustered, as they represent series of measurements that stem from different individuals. A multilevel approach to analyze these data takes into account clustering and can therefore accommodate these data and provides flexible tools to investigate within-subject phenomena, such as responses to stressors or rewarding experiences (see [9]). Data will be analyzed with a software that allows for the simultaneous modeling of within-subject and between-subject aspects of the data, and the examination of associations among individual difference variables, and individual differences in within-subject parameters (e.g., Mplus 7.3 (Muthen & Muthen, [55]-2012) or HLM software). To correlate these measures with the 18FDOPA uptake values, mean values for the AA measures will be computed.

##### Biological outcomes

Regarding the saliva samples, we will compute the cortisol awakening response (CAR) and alpha-amylase awakening response as well as daily profiles using an area under the curve approach. The CAR resp. AA awakening and the daily profiles will be used as biological indexes of stress reactivity in everyday life. The outcome is the difference between pre-test and post-test and the comparison between the MORE group and the waitlist group.

### Safety analysis

Safety analysis includes the report and observation of potential side-effects of the MORE intervention. Should side-effects occur, we will analyze the characteristics of the participants concerned.

### Interim analyses {21b}

Interim analyses will include pre-test comparisons (healthy controls versus FM) on the main and secondary outcomes after completion of the first MORE group as well as exploratory pre-post comparisons on the main outcomes to control whether methodological adjustments have to be made. Data of the pre-test comparisons (healthy controls versus FM) after completion of the second MORE group will be used for first data presentation and if possible publication of pilot data.

### Methods for additional analyses (e.g., subgroup analyses) {20b}

In an exploratory way, we will perform exploratory correlational analyses between the changes observed on our measures with the changes observed in 18F-DOPA influx to analyze the relationship between the neural and psychological mechanisms of change.

### Methods in analysis to handle protocol non-adherence and any statistical methods to handle missing data {20c}

Using an intent-to-treat analysis strategy allows to include all participants having completed the pre-test measures in case of drop-outs. In case of drop-outs, last observations will be carried forward.

### Plans to give access to the full protocol, participant-level data, and statistical code {31c}

Full protocol, anonymous participant-level data, and statistical code are available from the corresponding author upon request.

## Oversight and monitoring

### Composition of the coordinating center and trial steering committee {5d}

The coordinating center is at the University of Fribourg and the trial steering committee is led by CMS and KL. Regular meetings are taking place between (1) the study staff involved in the recruitment, screening, and conductance of the study on a weekly basis (online meetings) and (2) all investigators

### Composition of the data monitoring committee, its role, and reporting structure {21a}

Monitoring visits at the investigator’s site prior to the start and during the course of the study will help to follow up the progress of the clinical study, to assure the utmost accuracy of the data, and to detect possible errors at an early time point. The sponsor organizes professional independent monitoring for the study. All original data including all patient files, progress notes, and copies of laboratory and medical test results will be available for monitoring. The monitor will review all or a part of the eCRFs and written informed consents. The accuracy of the data will be verified by reviewing the above referenced documents. The investigator’s site will collaborate with the Appletree CI group.

### Adverse event reporting and harms {22}

During the entire duration of the study, all serious and non-serious adverse events (SAEs) that may be causally related to the study intervention are collected and documented in source documents. Reportable events are recorded in the case report form (CRF). Study duration encompassed the time from when the participant signs the informed consent until the last protocol-specific procedure has been completed including a safety follow-up period of 12 weeks (see follow-up measures). The recording of serious and non-serious adverse event (SAE and AE) information includes the time of onset, duration, resolution, action to be taken, assessment of intensity, and relationship with study treatment. Participants will be asked about health problems, mood worsening, and negative thoughts as well as any emergency medical or hospitalization that could have happened between 2 study visits at each visit, beginning at pre-test. Participants who prematurely stop the study will be asked for adverse events at the time, when they stop at the end of the study visit.

### Frequency and plans for auditing trial conduct {23}

A quality assurance audit/inspection of this study may be conducted by the Cantonal Ethics Committee (CEC). The quality assurance auditor/inspector will have access to all medical records, the investigator’s study-related files and correspondence, and the informed consent documentation that is relevant to this clinical study.

The investigator will allow the persons being responsible for the audit or the inspection to have access to the source data/documents and to answer any questions arising. All involved parties will keep the patient data strictly confidential.

### Plans for communicating important protocol amendments to relevant parties (e.g., trial participants, ethical committees) {25}

Substantial protocol amendments (significant changes) are only implemented after approval of the CEC. Under emergency circumstances, deviations from the protocol to protect the rights, safety, and well-being of human participants may proceed without prior approval of the sponsor and the CEC. Such deviations shall be documented and reported to the sponsor and the CEC as soon as possible. A list of substantial amendments is also available on www.swissethics.ch.

## Dissemination plans {31a}

After the statistical analysis of this trial, the sponsor will make every endeavor to publish the data in a peer-reviewed medical or psychological journal. In addition, preliminary data (from interim analyses) will be presented in scientific meetings and if possible published. Authorship for all investigators and associated investigators, no intended use of professional writers. The trial results will be published as peer-reviewed scientific papers and poster or oral presentations in conferences. All data and protocol will be available beginning 3 months and ending 3 years after the publication of the results. The trial data will be available from the corresponding author upon reasonable request.

## Discussion

Among chronic pain conditions, FM is a frequent and very disabling disease, which is still poorly understood and difficult to treat. This project aims to investigate if the mindfulness-based MORE intervention is able to restore the DA function in FM patients, in particular with regard to DA responses to reward, and to reduce pain and mood symptoms. More specifically, we expect the FM participants to show altered DA responses to reward before MORE compared to healthy controls, and we expect these alterations to be reduced after the MORE intervention compared to a non-intervention control group. Building on a previous project of our group pointing to a dysfunction of the DA system in FM [49], we will extend here the understanding of the role of DA in FM and investigate the potential of a non-pharmacological intervention to decrease pain and ameliorate mood in FM as well as to induce changes in the central DA function. With regard to the clinical effects of MORE, [26, 31] found a medium effect size of the intervention on pain severity. We used their effect size to calculate the size of our sample for the primary outcome of DA responses to monetary reward and are confident that the use of a multi-modal design combining clinical and AA measures will permit a better detection of the effects. At a clinical level, the use of standardized outcomes recommended for the study of FM will allow us to target clear variables for changes associated with the intervention. Using a non-intervention control design instead of a design with an active control group might increase the effects as we will not control for placebo effects. However, the justification is based on ethical and methodological reflections that have been discussed in the design section, and constitute for this sample with these specific measures is the state-of-the-art. We are aware that proper AA analyses need larger samples; however, the sample sizes calculated correspond to the main outcome and other studies in FMS studies and will allow for exploratory micro-analyses.

At a scientific level, this project is highly innovative as it will integrate in vivo brain imaging of the DA system in response to motivational stimuli with AA measures in everyday life, allowing for the association between daily life momentary affective states and self-reported reward sensitivity with DA transmission elicited by rewards in an experimental setting. In addition, the investigation of potential changes of the DA reactivity after a mindfulness-based intervention reveals crucial information concerning the potential plasticity of the DA system. At a clinical level, MORE is a manualized therapy comparably easy to teach and implement. It could therefore be used in several clinical settings and at a larger scale. The integration of a large network of outpatient clinics in this project will allow for a rapid dissemination of the methods if MORE shows significant results. The integration of AA measures as outcome measures for the MORE intervention is not only innovative, but it will also allow a transfer of the intervention-related training effects in the everyday life of the participants so to increase the ecological validity of our results. Finally, we are confident that our results will both bring a better understanding of FM as well as integrating neuroscience findings into treatment development by targeting neural mechanisms underlying FM. However, some challenges need to be addressed. We have the following reasons to justify the use of a design with a non-intervention group and not an active control group. First, placebo group interventions in the psychological setting, often lacking credibility, can elicit so-called resentful demoralization [61], leading to increased drop-out risk in these groups. With regard to the study of [26, 31] indicating more than 20% drop-out during the treatment, it is important to minimize this risk. From an ethical point of view, it is questionable to provide a non-efficacious treatment to patients having a long history of such treatments as it is unfortunately the experience of many FM patients. Because of the symptomatology (mobility difficulties due to pain symptoms, fatigue, etc.), it is difficult for patients to commit to a treatment, so it would be a waste of both time and energy for them to come to our centers for pseudo-intervention. From a methodological point of view, support group interventions are problematic: they will not be comparable across time and different groups since the interaction between the participants and the therapists cannot be fully standardized. This is particularly complicated when the intervention is given in 3 different centers, and might impair the validity of such a control intervention. For these reasons, RCT list designs can be justified to assess the effects of psychological interventions in populations with somatic disorders. To minimize bias, we will use only standardized questionnaires to assess the secondary outcomes as recommended by the international organized network aiming at improving outcome measures in rheumatology (OMERACT). Furthermore, clinical outcomes and especially self-reported pain could be influenced by knowledge of an assigned intervention. Recruitment in general is a challenge for large clinical trials with chronic pain patients, but we have faced this challenge previously and have developed effective strategies for recruitment [49, 50]. For this more ambitious trial, recruiting multi-centrically participants in the German- and the French-speaking parts of Switzerland will allow to complete the study. In addition, we have created a large network of outpatient clinics and practices with our project partners at the different centers, who will directly support the study with recruitment. With regard to the medication, opioids should not be too much of a concern, given low efficacy in these patients, and relatively restrictive prescription practices in Switzerland for this population.

Another barrier of recruitment is going to be the requirement of randomization into immediate or delayed groups. As such, participants will have to make themselves available for program dates over a period of 5 months, in case they are randomized to the waitlist groups. Logistically managing simultaneous baseline and follow-up measures can be challenging. Another issue is drop-outs or missing data. By using an online format for the questionnaires, we aim to eliminate incomplete datasets. Due to the forced-answer format of the questions in the questionnaires, no missing data is to be expected. In case of missing data, the last observation will be carried forward. Drop-outs will be replaced in order to reach the targeted number of complete participants in the study. To evaluate the influence of drop-outs, analyses will be first performed with complete datasets only and compared to results of intent-to-treat analyses where all participants are included. Lastly, most clinical outcomes are patient-reported and related to pain, which are heavily influenced by knowledge of assigned intervention so this could possibly influence the outcomes.

## Trial status

The protocol number version 6, dated 30.03.2021. Recruitment started in June 2021 and will approximately be finished in October 2024. The protocol was registered under ClinicalTrials.gov NCT 044515664. Registered on 3 July 2020. The trial was prospectively registered.

## Abbreviations

AA: Ambulatory assessment
AE: Adverse event
BDI: Beck Depression Inventory
BPI: Brief Pain Inventory
CAR: Cortisol awakening response
CBT: Cognitive behavioral therapy
CEC: Cantonal Ethics Committee
CERQ: Cognitive Emotion Regulation Questionnaire
CHUV: University Hospital Lausanne
CNS: Central nervous system
CPRS: Complex regional pain syndrome
CRF: Case report form
DA: Dopamine
eCRF: Electronic case report form
FM: Fibromyalgia syndrome
FIQ: Fibromyalgia Impact Questionnaire
FMS: Fibromyalgia syndrome
fMRI: Functional magnetic resonance imaging
GCP: Good clinical practice
HIPAA: Health Insurance Probability and Accountability Act
MDD: Major depressive disorder
MINI: Mini-International Neuropsychiatric Interview
MORE: Mindfulness-Oriented Recovery Enhancement
MOS: Medical outcomes study sleep scale
Nacc: Nucleus accumbens
NA: Negative affect
PI: Principal investigator
RCT: Randomized controlled trial
OMERACT: Outcome Measures in Rheumatology Clinical Trials
PA: Positive affect
PET: Positron emission tomography
PDI: Pain Disability Index
PSQI: Pittsburgh Sleep Quality Index
QoL: Quality of life
RedCAP: Research Electronic Data Capture
SAE: Serious adverse event
STAI: State-Trait Anxiety Inventory
SOP: Standard operating procedure
VRS: Visual analog scale
WHOQOL: World Health Organization Quality of Life Questionnaire
WI: Working instruction
WPI: Widespread pain index
18-F DOPA: 18-F fluorodopa
